# A novel asymmetrical anti-HER2/CD3 bispecific antibody exhibits potent cytotoxicity for HER2-positive tumor cells

**DOI:** 10.1186/s13046-019-1354-1

**Published:** 2019-08-14

**Authors:** Shengnan Yu, Jing Zhang, Yongxiang Yan, Xudong Yao, Lijuan Fang, Hui Xiong, Yang Liu, Qian Chu, Pengfei Zhou, Kongming Wu

**Affiliations:** 1 Department of Oncology, Tongji Hospital of Tongji Medical College, Huazhong University of Science and Technology, 1095 Jiefang Avenue, Wuhan, 430030 People’s Republic of China; 2 Wuhan YZY Biopharma Co., Ltd, Biolake, C2-1, No.666 Gaoxin Road, Wuhan, 430075 People’s Republic of China; 3 Department of Orthopedics, Tongji Hospital of Tongji Medical College, Huazhong University of Science and Technology, Wuhan, 430030 China

**Keywords:** Bispecific antibody, HER2, CD3, Breast cancer, Knobs-into-holes

## Abstract

**Background:**

Human epidermal growth factor receptor 2 (HER2) is overexpressed in multiple cancers, which is associated with poor prognosis. Herceptin and other agents targeting HER2 have potent antitumor efficacy in patients with HER2-positive cancers. However, the development of drug resistance adversely impacts the efficacy of these treatments. It is therefore urgent to develop new HER2-targeted therapies. Bispecific antibodies (BsAbs) could guide immune cells toward tumor cells, and produced remarkable effects in some cancers.

**Methods:**

A BsAb named M802 that targets HER2 and CD3 was produced by introducing a salt bridge and knobs-into-holes (KIHs) packing into the structure. Flow cytometry was performed to determine its binding activity and cytotoxicity. CCK-8, Annexin V/PI staining, western blotting, and ELISA were utilized to study its effect on cell proliferation, apoptosis, the signaling pathways of tumor cells, and the secretion of cytokines by immune cells. Subcutaneous tumor mouse models were used to analyze the in vivo antitumor effects of M802.

**Results:**

We generated a new format of BsAb, M802, consisting of a monovalent unit against HER2 and a single chain unit against CD3. Our in vitro and in vivo experiments indicated that M802 recruited CD3-positive immune cells and was more cytotoxic than Herceptin in cells with high expression of HER2, low expression of HER2, and Herceptin resistance. Although M802 showed weaker effects than Herceptin on the PI3K/AKT and MAPK pathways, it was more cytotoxic due to its specific recognition of HER2 and its ability to recruit effector cells via its anti-CD3 moiety.

**Conclusions:**

Our results indicated that M802 exhibited potent antitumor efficacy in vitro and in vivo. M802 retained the function of Herceptin in antitumor signaling pathways, and also recruited CD3-positive immune cells to eliminate HER2-positive tumor cells. Therefore, M802 might be a promising HER2 targeted agent.

**Electronic supplementary material:**

The online version of this article (10.1186/s13046-019-1354-1) contains supplementary material, which is available to authorized users.

## Background

Bispecific antibodies (BsAbs) have been considered as effective therapeutic candidates for the treatment of cancers and are currently in various stages of clinical development [1]. In particular, BsAbs have been designed to target tumor cells and effector cells simultaneously, triggering the cytotoxicity of effector cells toward tumor cells [2]. Initially, BsAbs were produced by hybrid-hybridoma technology [3] or directed-chemical coupling [4, 5]. However, both technologies are difficult to be used in manufacture, because of their low efficiency of heterodimer formation and high cost.

With the advent of recombinant DNA technology, various forms of bispecific antibody fragments, including diabodies, tandem scFv, single-chain diabodies, and derivatives thereof, could be produced by genetic engineering [6, 7]. Some BsAb formats, such as bispecific T-cell engagers (BiTE) [8–10] and Dual-affinity Re-Targeting (DART) [11], have short half-lives in vivo and lack antibody-dependent cell-mediated cytotoxicity (ADCC) effect due to their low molecular weight and the absence of IgG Fc domain, although they exhibit better tumor-penetration ability. The scFv format is a useful platform for antibody engineering [12] and some BsAbs were constructed based on this technology [13]. There are other formats of BsAbs, such as strand exchange engineered domain (SEED) [14] and CrossMab [15], but their downstream process development is challenging because it is difficult to distinguish homodimers from heterodimers.

Several strategies have been developed to improve the heterodimerization of BsAbs by genetic modifications of the CH3 chains, such as disulfide bonds [16], knobs-into-holes (KIHs) [17], and salt bridges, which were equal to electrostatic steering effects [18, 19]. KIHs are preferred over Fc fragment modification due to their high efficiency of heterodimer formation (> 85%) [20]. Application of salt bridge on bispecific antibodies was first reported by Paul Carter [18]. It provided an alternative method to efficiently produce heterodimer through ionic bonds of oppositely charged residues along the interface. Amgen Inc. reported a similar platform that changed the positive charges to negative (e.g. K392D, K409D) on one Fc fragment and the negative charges to positive (e.g. D356K, D399K) on the opposite Fc fragment, which resulted in the intracellular assembly of heterodimers with an efficiency of more than 95% [19].

The mispairing between light chains and heavy chains in BsAbs is still problematic. In Genentech’s study, BsAbs utilizing the KIHs and common light chain could be produced with large quantities in bacteria and were active in vivo [21]. Pfizer’s study shows that two antibodies with mutations on their heavy chains could be expressed and purified separately, then mixed together under appropriate redox conditions, which resulted in formation of a stable BsAb with high yield [22]. Genmab’s study is very similar with Pfizer’s and based on the mechanism called “controlled Fab-arm exchange (cFAE)” [23]. A common light chain can solve the problem of mispairing between light chains and heavy chains. However, the common light chain is hard to be screened and BsAbs with common light chain were difficult to be analyzed and purified by standard methods due to the high similarity between the heterodimer and homodimer.

Therefore, it is necessary to design BsAbs that are more stable, easier to analyze, and produce with low toxicity and immunogenicity for patients. In this study, we describe a new format of BsAb which was constructed using a salt bridge and KIHs mutations in the CH3 domain of the Fc fragment. Besides, we introduced an asymmetrical structure which contains a monovalent unit and a single chain unit to avoid the need for a common light chain. We designed this new BsAb named M802, of which the monovalent unit specifically binds to HER2 and the single chain unit binds to CD3. Based on the different molecular weights and isoelectric points caused by the structure and mutations between heterodimer and homodimer, we could easily purify M802 by ion-exchange chromatography and evaluate the purity by SDS-PAGE.

HER2, a 180 KDa transmembrane glycoprotein, is expressed on the surface of diverse tumor cells, including 20 to 30% of breast cancers [24], 20% of gastric cancers [25], and 12 to 15% of gallbladder cancers [26]. Amplification of the *HER2* gene or overexpression of the HER2 protein plays an important role in the development of malignant cancers [27], and HER2 is considered as a crucial target for antitumor treatment. In 1998, trastuzumab (trade name Herceptin), a recombined humanized anti-HER2 monoclonal antibody, was approved by Food and Drug Administration (FDA) for the treatment of HER2-positive advanced breast cancer [28]. However, approximately 70% of patients develop resistance to Herceptin, and some patients present with primary resistance [29]. There is an urgent need to develop new treatments targeting HER2 for this part of patients [30]. It is well known that CD3 is a surface marker of T lymphocytes, which is important in killing tumor cells [31]. It is an ideal strategy using M802 to manipulate CD3-positive T cells to eliminate HER2-positive tumor cells. In our study, both in vitro and in vivo results demonstrated that M802 was more cytotoxic for HER2-positive tumor cells than Herceptin through recruiting CD3-positive immune cells.

## Materials and methods

### BsAb construction, transfection, and purification

Primer sequences for BsAb construction are supplied in Additional file 1: Table S1. The anti-HER2 monovalent unit and the anti-CD3 single chain unit of M802 were from Herceptin and L2K, respectively. The sequence of Herceptin (PDB No.1N8Z) was obtained from the RCSB PDB protein data bank website, the protein sequence was reversely translated into the DNA sequence on the NCBI website, and this sequence was then used as a template for PCR amplification. The gene encoding the anti-CD3 single chain unit was reversely translated from the referenced protein sequences of L2K (US20070123479 sequence No.2). The Fc fragment of scFv-Fc was the human IgG1 Fc fragment (GenBank accession No.AF150959). All PCR products were first inserted into the T-vector pEASY-T1 (TransGene, China) and verified by sequencing, followed by doubled digested. The expression vectors included pcDNA 3.1/Hygro (+)-Herceptin-Light-chain, pcDNA3.1/Hygro (+)-L2K-Single-chain, and pcDNA (−)-Herceptin-Heavy-chain. The gene encoding the alternative molecule targeting human HER2 and murine CD3 (GenBank, GI:841159/841161) was cloned into the expression plasmid pcDNA3.4-TOPO AMC3-scFv-Fc. The mutations on the CH3 domains of the Fc fragment included the T366 W-Y407A (KIHs pair), the L368R-K409D (ionic bond “salt bridge”), and D399K-K392D (second salt bridge). All vectors and mutants were verified by sequencing (Huada Gene, China).

The plasmids were transfected into 293F cells (Invitrogen) using the Endofree Plasmid Giga Kit (Qiagen, 12,391) according to the manufacturer’s protocol. The cell culture supernatant was collected and purified through a Sepharose Fast Flow protein A affinity chromatography column (GE), Fab Affinity KBP Agarose High Flow Resin (ACROBio systems), and SP cation-exchanged chromatography column (GE). The purified proteins were analyzed by SDS-PAGE and Coomassie blue staining.

### Thermal challenge assay

Aliquoted antibodies (0.5 mg/mL) were heat-treated in PCR strip tubes for 60 min in a thermal cycler (ABI PCR system 9700), and then allowed to bind to SK-BR-3 cells. PE-labeled goat anti-human IgG was used as the secondary antibody. The stained cells were analyzed using flow cytometry (FC500, Beckman). For analysis of thermal gradients, the data were fit to a model with a sigmoidal dose-response curve and variable slope using GraphPad Prism 6 software. The mid-point of each thermal denaturation curve is referred as the T_50_, which are not construed as being equivalent biophysically.

### Charge variants identification

Capillary isoelectric focusing was performed to identify charge variants of BsAbs using the BECKMAN PA800 *plus*.

### Molecular weight determination by LC-MS

Reverse-phase (RP) HPLC was performed using an ultrahigh-performance system (Acquity UPLC H-CLASS BIO, Waters) that was coupled to a Quadrupole Time-of-Flight (QTOF) mass spectrometer (XEVO G2 QTOF, Waters). The G2 QTOF was operated in ESI positive ion mode (capillary voltage: 3000 V, sample cone voltage: 30 V).

### Surface plasmon resonance assay

A surface plasmon resonance (SPR) assay was used to analyze binding of each BsAb to the corresponding ectodomains of hHER2 and hCD3 using a ProteOnTm XPR36 biosensor (Bio-Rad). In this dual-binding assay, HER2 and CD3 antigens were vertically immobilized on different channels (L4 and L5) in a GLC chip using the amine coupling kit at an immobilization level of 1000 resonance units (RU), as recommended by the manufacturer. The recombinant HER2 and CD3 antigens were produced by Wuhan YZY Biopharma Co., Ltd..

### Cell lines and antibodies

Cell lines of human breast cancer (SK-BR-3, BT-474, and MDA-MB-231), gastric cancer (NCI-N87), T-cell leukemia (Jurkat), and Human Embryonic Kidney cell 293 (HEK 293) were purchased from the China Center for Type Culture Collection (CCTCC). The Herceptin-resistant human breast cancer cell line (JIMT-1) was purchased from Deutsche Sammlung von Mikroorganismen und Zellkulturen (DSMZ). JIMT-1 and MDA-MB-231 cells were cultured in DMEM (Gibco), and all other cells were cultured in RPMI 1640 (Gibco) with 10% fetal bovine serum (Gibco). All cells were maintained at 37 °C in a 5% (vol/vol) CO_2_ humidified incubator.

The B16 Mouse melanoma cell line (TCM2) was purchased from the Type Culture Collection of the Chinese Academy of Science (Shanghai, China). The B16-HER2 cell line was established by transfection with a vector (pcDNA3.4-TOPO, Invitrogen, A14697) that contained the full-length cDNA of hHER2 (acquired by RT-PCR from the mRNA of NCI-N87).

The BsAbs used in this study include M802 targeting hHER2 and hCD3, M806 targeting hHER2 and mCD3, MCO101 targeting fluorescein and hCD3, and MCO106 targeting fluorescein and mCD3. In addition, these BsAbs were constructed on the basis of a monovalent unit and a single chain unit, so this type of BsAb is collectively called MSBODY.

### Cell binding assay

SK-BR-3 cells were incubated with serially diluted M802 or Herceptin for 1 h at room temperature, and then with PE-conjugated anti-human IgG Fc (secondary antibody) for 30 min in the dark. The binding activity was measured by flow cytometry. The same method was used to measure the affinity of M802 and L2K to Jurkat cells. To measure the co-binding ability of M802, HER2-positive SK-BR-3 cells were labeled with 2.5 μM CFSE (Invitrogen) and CD3-positive Jurkat cells were labeled with 0.2 μM PKH26 (Sigma). Cells were then washed three times and mixed at a ratio of 1:1. Mixtures were incubated with serially diluted antibody (0 to 10 μg/mL) for 30 min at 37 °C in a 96-well round bottom plate (Corning). Co-binding was measured using flow cytometry and indicated as the percentage of cells in the upper right quadrant of an FL1 vs. FL2 scatter plot, representing the CFSE-PKH26-double-positive population.

### In vitro cytotoxicity assay

Peripheral blood mononuclear cells (PBMCs) of healthy donors were used as effector cells to determine the in vitro efficacy of the M802. These cells were isolated from blood samples by Ficoll-Hypaque (Sigma) density gradient centrifugation according to the manufacturer’s instructions. To assess the strength of redirected lysis by M802, PBMCs were incubated with M802 or control antibodies for 30 min at room temperature, the mixture was washed three times with 1% FBS-PBS to remove excess antibodies, and the coated PBMCs were incubated with SK-BR-3 target cells at different E:T ratios (1:1 to 20:1), and then lysis was measured after 48 h via FACS by nuclear uptake of propidium iodide (PI) (Sigma). After determining a suitable E:T ratio, the bioactivity M802 was determined by a FACS-based in vitro cytotoxicity assay. The target cells include HER2-positive breast cancer cell lines (SK-BR-3 and BT-474), Herceptin-resistant cell line JIMT-1, HER2-positive gastric cancer cell line (NCI-N87), HER2-negative breast cancer cell line (MDA-MB-231), and HER2-negative cell line (HEK-293). A total of 2.0 × 10^4^ target (T) cells labeled with CFSE were co-incubated with effector (E) cells at an E:T ratio of 5:1 in the presence of serially diluted M802 or control antibodies in 96-well flat-bottomed plates (Corning) for 48 h at 37 °C. Cells were collected and stained with PI for FACS analysis.

### Cell viability assay

Cells were seeded at 2.0 × 10^4^ per well into 96-well plates. After adhesion, cells were treated with different concentrations of M802 or Herceptin. The CCK-8 cell proliferation kit (Dojindo, Kumamoto, Japan) was used to measure cell viability on 6 consecutive days. For each test, 10 μL of the CCK-8 reagent was added to the cells, the cells were incubated at 37 °C for 3 h, and the absorbance at 450 nm was then measured using a microplate reader (Molecular Devices).

### Apoptosis assay

Cells were seeded in triplicate into 24-well plates at a concentration of 2.0 × 10^5^ cells/well, and then treated with 3 different concentrations of M802 or Herceptin. After 48 h at 37 °C and 5% CO_2_, cells were dissociated with trypsin/EDTA (Gibco), washed twice with cold PBS, and then labeled with Annexin V/PI (BioLegend) as described previously [32].

### Western blotting

Protein was extracted from cultured cells using a lysis buffer (Beyotime) with phosphatase and protease inhibitors (Boster) as described previously [33]. A total of 30 μg protein was separated by SDS-PAGE and then transferred to polyvinylidene fluoride (PVDF) membranes (Immobilon-PSQ). The membranes were blocked with 5% Bovine Serum Albumin (BSA) (Bovogen), probed with primary antibodies overnight at 4 °C, and then incubated with the relevant secondary antibody for 2 h at room temperature. Electrochemiluminescence (ECL, BioRad) with a detection reagent (Advansta) was used to detect signals. The antibodies used in western blotting included anti-Akt (CST, 9271S), anti-pAkt (CST, 12694S), anti-Erk (CST, 9102S), anti-pErk (CST, 9106S), anti-cyckinD1 (Abcam, ab134175), anti-caspase-3 (Abcam, ab32351), anti-cleaved-caspase-3 (CST, 9664S), anti-P21 (Abcam, ab109520), anti- P27^kip1^ (Abcam, ab32034), anti-β-actin (Boster, BM5422), anti-rabbit-IgG-HRP (CST, 7074S), and anti-mouse-IgG-HRP (CST, 7076P2).

### T cell activation and cytokine release assay

PBMCs from healthy donors were co-cultured with target cells (SK-BR-3) and different concentrations of antibodies (0.0001 to 10,000 ng/mL) for 48 h in 96-well flat-bottomed well plates. At the point of detection, cells were collected and stained with anti-CD3-FITC (BioLegend, 300,306) and anti-CD25-APC (BioLegend, 302,610) and anti-CD69-PE (BioLegend, 310,906) for 30 min at room temperature in the dark. T-cell activation was determined using flow cytometry (FC500, Beckman). Cell-free supernatant was collected to quantify the BsAb-induced cytokines release. Cytokines including interleukin (IL)-2, IL-6, IFN-γ and TNF-α were measured with ELISA kits (R&D Systems) according to the manufacturer’s instructions.

### Animal studies

The protocols of all animal experiments were approved by the Institutional Animal Care and Use Committee of Tongji Hospital of Huazhong University of Sciences and Technology. Female C57BL/6, NOD/SCID and BALB/C mice (7 to 8 weeks old), were purchased from Beijing HFK Bioscience Co. Ltd. and housed in a barrier unit of the Central Laboratory in Hubei Provincial Food and Drug Safety Evaluation Center. PBMCs were co-cultured with anti-CD3 monoclonal antibody, IFN-γ, IL-2, and IL-1α for 14 days to obtain activated PBMCs, also known as the cytokine-induced killer (CIK) cells.

5.0 × 10^6^ NCI-N87 cells and 5.0 × 10^6^ CIK cells were mixed and subcutaneously inoculated into the right dorsal flank of 40 NOD/SCID mice. Within 2 h, the mice were randomly divided into two experimental groups and three control groups (8 per group). Mice in the experimental groups received M802 (2 mg/kg or 1 mg/kg) by intravenous (i.v.) bolus injection into the lateral tail vein. Mice in the control groups received Herceptin (2 mg/kg), MCO101 (2 mg/kg), or saline. Each treatment was repeated on day 2 and day 4.

B16-HER2 murine model was established by subcutaneous inoculation of 3.0 × 10^6^ B16-HER2 cells into the right dorsal flank of C57BL/6 mice. On each of the next 3 days, mice received i.v. bolus injections into the tail vein of 0.125 mg/kg M806, 0.0625 mg/kg M806, 4 mg/kg Herceptin, 0.1 mg/kg MCO106, or saline (8 per group). Before and after treatment, the level of lymphocytes in peripheral blood was monitored. Tumor volumes (mm^3^) were measured every 3 days using a digital caliper and the formula: ½ × (length × width^2^). Mice were euthanized when tumor volume was 2000 mm^3^ or when the study ended. Tumors were isolated and fixed in 10% formalin for immunohistochemical (IHC) staining using anti-mouse CD3 (Abcam, ab16669), anti- mouse CD4 (Abcam, ab183685), and anti- mouse CD8 (Abcam, ab217344).

BALB/C mice (6 per group) were received i.v. bolus with M802 or Herceptin (8 mg/kg). Serum samples were collected at different timepoints to determine pharmacokinetic (PK) parameters using ELISA kits (BioLegend). The systemic clearance (Cl), maximum serum concentration (C_max_), and serum elimination half-life (T_1/2_, β) were estimated using DAS, version 2.1.1 (Bontz Inc., Beijing, China).

### Statistical analysis

Statistical analysis was performed using SPSS16.0 (SPSS Inc., Chicago, IL) and Prism 6 (GraphPad Software Inc., La Jolla, CA). Statistical analysis between groups were calculated using two-sided Student’s *t*-test. All data are presented as means ± SD. *P* value < 0.05 was considered statistically significant.

## Results

### Generation and characteristics of M802

**Fig. 1:**
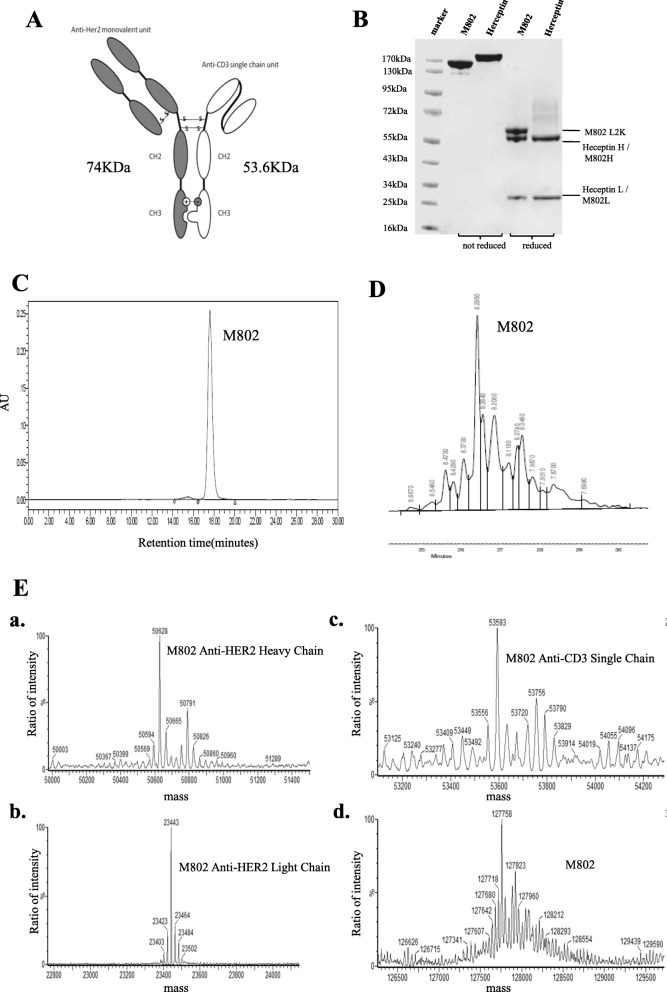
Characteristics of M802. (**a**) Schematic diagram of M802, which consisted of a monovalent unit and a single chain unit. The monovalent unit consisted of M802H and M802 L conjugated by a disulfide bond. The single chain unit was L2KVH-linker-L2KVL-Fc structure. Two disulfide bonds were formed between the monovalent unit and the single chain unit. (**b**) SDS-PAGE of M802 from 293F cells purified by protein A affinity and ion-exchange chromatography. In the reducing SDS-PAGE, the single chain unit and heavy chain of the monovalent unit (M802H) showed slightly different electrophoretic mobility. (**c**) Analytical size-exclusion chromatograms of purified M802 described in (**b**), and the purity of this sample was more than 98%. (**d**) Isoelectric focusing of Herceptin and M802. For M802, two main charge variant bands that migrated above PI 8.0 and below Herceptin. (**e**) Mass spectrum for molecular weight determination of the intact BsAb and chains under reducing conditions. The molecular weight of M802H was 50.628 KDa, M802 L was 23.443 KDa, L2K single chain was 53.539 KDa, and intact M802 was 127.758 KDa

M802 has two units: a monovalent heavy chain/light chain pair and a single chain unit (Fig. 1a). SDS-PAGE under reducing conditions showed three bands which indicated that M802 consists of three peptides of heavy chain, light chain and single chain (Fig. 1b). SEC indicated the purity of M802 was more than 98% (Fig. 1c). Asymmetrical isoelectric point (pI) was a characteristic of M802. The monomer or homodimer of monovalent unit’s pI was about 7.8, and the monomer or homodimer of single chain unit’s pI is about 8.9, while M802’s pI was about 8.5 (Fig. 1d). The unique properties of M802 were used for purification of this BsAb using ion-exchange chromatography after protein A purification.

Under reducing conditions, the molecular weight (Mw) of the monovalent unit was about 74 KDa (heavy chain 50.6 KDa + light chain 23.4 KDa; Fig. 1e, a and b), and the single chain unit was about 53.6 KDa (Fig. 1e, c). The Mw of the homodimer of monovalent unit was 148 KDa, and the Mw of the homodimer of the single chain unit was 107.2 KDa, while the heterodimer (M802) had a Mw of 127.7 KDa (Fig. 1e, d). This asymmetrical BsAb avoids a common light chain, and impurities (such as homodimers) are easy to remove because of their different Mws. Except for a few mutations, the Fc fragments of M802 were identical to those of Herceptin, which was a part of constant region of human IgG1. The mutations in the Fc fragments of M802 were based on a salt bridge and KIHs. The specific modifications were T366 W, K392D, and K409D on the monovalent unit and L368R, D399K, and Y407A on the single chain unit.

The scFv of the single chain unit was built into the VH-VL orientation, and joined in frame via a (Gly4Ser)3 linker to the amino terminal region of the VH domain. The intrinsic stability of the scFv moiety is an important factor in determining the quality of an antibody [34]. The result of thermal challenge assay of M802 showed that the stability of the unit specific to HER2 was close to that of Herceptin. Similarly, the thermal stability of unit specific to CD3 was close to that of L2K (Additional file 1: Table S2). These results indicate that M802 has potential for use as a therapeutic BsAb because its stability at elevated temperatures was similar to that of the IgG1 antibody.

### Binding activity of M802

**Fig. 2:**
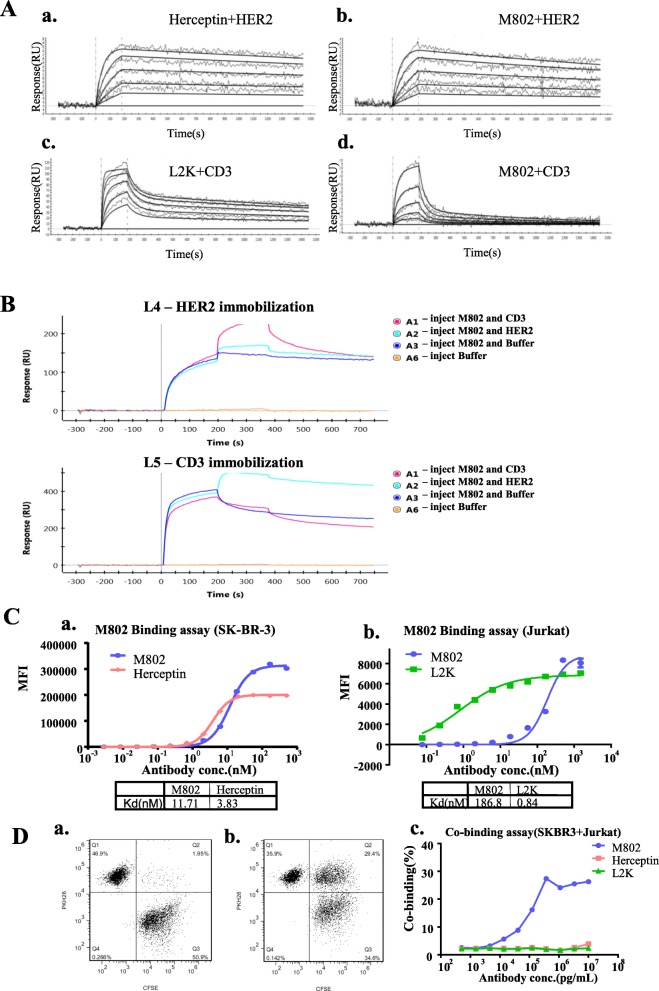
Binding properties of M802. (**a**) Kinetics of the binding of Herceptin and M802 to immobilized HER2 antigen and of L2K and M802 to immobilized CD3 antigen by SPR assay. M802 (0 to 50 nM) and other antibodies were injected over immobilized HER2 and CD3. (**a**) and (**b**) binding curves were analyzed using 1:1 L interaction model. (**c**) and (**d**) binding curves were analyzed using Two state interaction model. (**b**) Dual-binding activity of M802. The HER2 and CD3 antigens were vertically immobilized on two different channels (L4 and L5) in a GLC chip, and then 200 nM M802 was horizontally injected into channels A1, A2, and A3. Then 200 nM CD3 was injected into channel A1, 200 nM HER2 into channel A2, and PBST buffer into channel A3. (**c**) Cell binding activity of M802. (**a**) The affinity of M802 at the anti-HER2 moiety on SK-BR-3 cells. (**b**) The affinity of M802 at the anti-CD3 moiety on the Jurkat cells. (**d**) M802-mediated cell-to-cell association. The association of SK-BR-3 cells (stained with CFSE) with Jurkat cells (stained with PKH26) without M802 (**a**) or with M802 (**b**) was presented. Cells in the upper right quadrant of FL1 vs. FL2 scatter plot represented the double-positive population. (**c**) The dose dependence of association mediated by M802

We next examined the binding affinity of M802 using the SPR assay. The equilibrium dissociation constant (Kd) for binding of M802 to HER2 is 5.78 × 10^− 10^ M, similar to that of Herceptin (1.14 × 10^− 10^ M; Fig. 2a, a and b; Additional file 1: Table S3). The affinity of M802 binding to HER2 was close to Herceptin probably because of their identical format of variable regions. Previous research reported that affinity of a single chain unit is weaker than a monovalent unit [35], as also indicated in our results. The affinity of M802 binding to CD3 (Kd = 7.12 × 10^− 8^ M) was much weaker than that of the binding of L2K (Kd = 1.23 × 10^− 9^ M) (Fig. 2a, c and d; Additional file 1: Table S3). We also measured the dual binding affinity of M802 by immobilizing either HER2 or CD3. The soluble signal that resulted when M802 was bound to CD3 and HER2 demonstrated its dual binding affinity (Fig. 2b).

The cell binding activity of M802 was measured by flow cytometry. The results indicated that the affinity of M802 was about three times weaker than that of Herceptin (Fig. 2c, a) and significantly weaker than that of L2K (Fig. 2c, b). These results are consistent with the results of the SPR assay. In addition, the mechanism of M802 mediated T cells recruitment to cancer cells was investigated. Jurkat cells expressing CD3 were labeled with PHK26 and HER2 positive SK-BR-3 cells were labeled with CFSE. The proportion of double-positive cells was about 1.95% at the absence of M802 (Fig.2d, a). In contrast, at the presence of 10 μg/mL M802, up to 29.4% of the total cell population was double-positive in the results of M802-mediated cell-to-cell association (Fig. 2d, b). The enhanced level of cell-to-cell association mediated by M802 was also dose dependent. Neither Herceptin nor L2K was able to mediate the association at any concentration tested (Fig. 2d, c).

### M802 exhibited potent redirected lysis to HER2-positive tumor cells

**Fig. 3:**
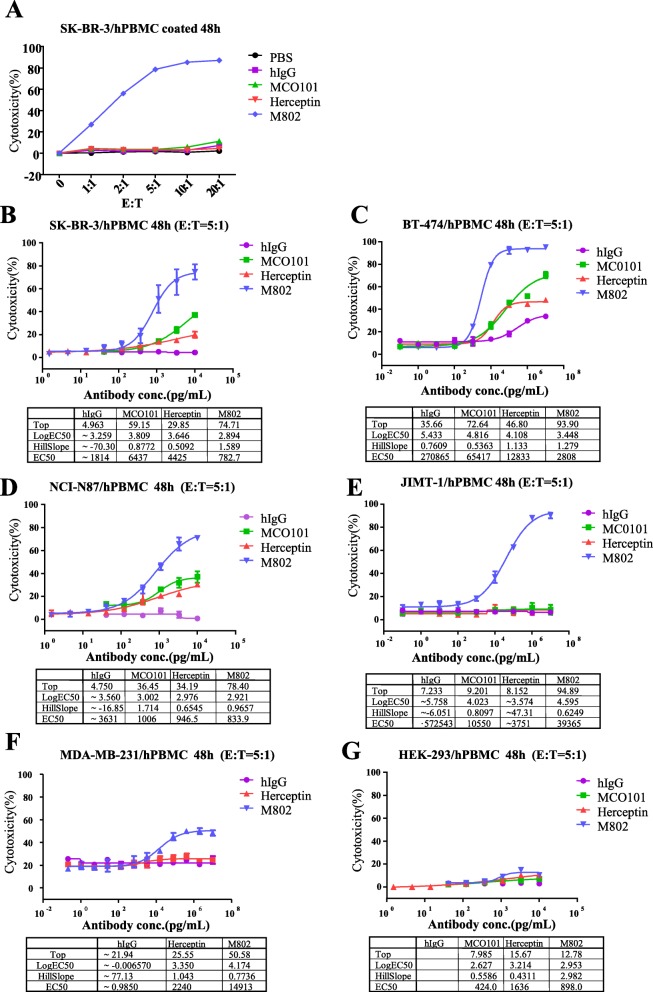
Cytotoxicity of M802 in vitro. (**a**) The coated PBMCs were incubated with SK-BR-3 target cells at different E:T ratios (1:1 to 20:1), and lysis was measured after 48 h via FACS, based on nuclear uptake of PI. The lysis rate of SK-BR-3 cells reached the maximum when PBMC coated with M802 antibody at the E:T ratio of 5:1 or more, but not with control antibodies. M802 mediated redirected lysis of human PBMCs to cancer cells expressing HER2. HER2-positive cancer cell lines SK-BR-3 (**b**), BT-474 (**c**) and NCI-N87 (**d**), HER2-negative breast cancer cell line MDA-MB-231 (**e**), Herceptin-resistant cell line JIMT-1(**f**) and non-cancer cell line (HEK-293 (**g**) were incubated in the presence of different concentrations of M802 with PBMCs at the E:T ratio of 5:1

Redirected lysis of SK-BR-3 cells only occurred when PBMCs were coated with the M802 antibody, not with control antibodies. M802 coated PBMCs showed cytotoxicity to cancer cells at different E:T ratios and the maximum lysis was reached at ratio of 5:1 or above. In contrast, the control antibodies showed almost no cytotoxicity (Fig. 3a).

We examined the M802 mediated cytotoxic effects via PBMCs on cancer cells with high and low expression of HER2. The target cells with different levels of HER2 (Additional file 1: Figure S1) were incubated in the presence of increasing concentrations of M802, with PBMCs at the E:T ratio of 5:1. M802 exhibited remarkable cytotoxic effects against SK-BR-3 cells (Fig. 3b), BT-474 cells (Fig. 3c), NCI-N87 cells (Fig. 3d), and JIMT-1 cells (Fig. 3e). However, M802 showed little cytotoxic effect on MDA-MB-231 cells because of their extremely low expression of HER2 (Fig. 3f). Herceptin and MCO101 only showed very limited cytotoxic effects on target cells at high concentrations. Our results indicated that BT-474 cells were quite sensitive to killing by PBMCs, and even human IgG showed certain cytotoxic effects at high concentrations. M802 treatment led to no significant lysis of non-cancerous cells (HEK-293), which indicated that the cell lysis mediated by M802 was specific. (Fig. 3g).

### M802 inhibited proliferation and promoted apoptosis of tumor cells

**Fig. 4:**
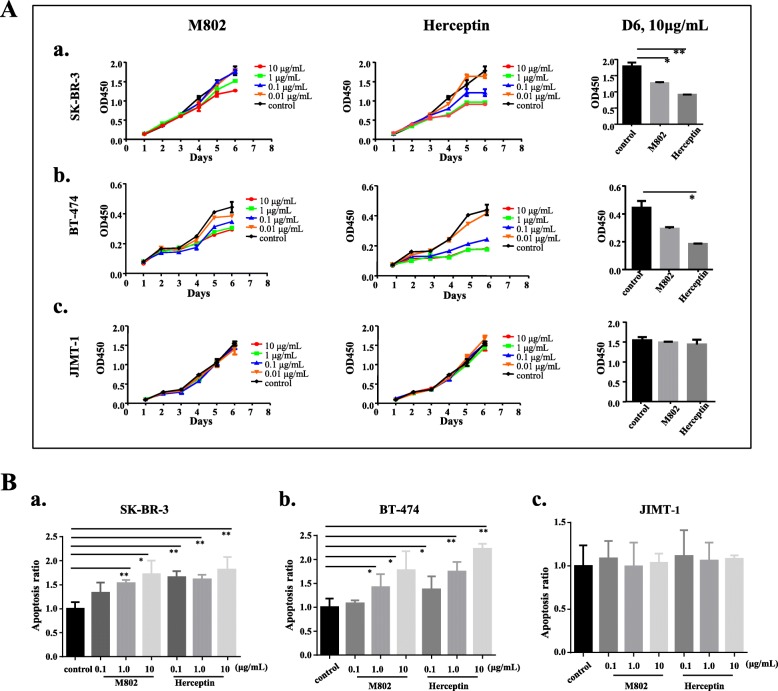
M802 inhibited proliferation and enhanced apoptosis of tumor cells in vitro*.* (**a**) M802 or Herceptin were incubated with SK-BR-3 cells (**a**), BT-474 cells (**b**), or JIMT-1 cells (**c**). After 48 h, cells were stained with Annexin V/PI and fluorescence was measured using flow cytometry. (**b**) Analysis of the fold-change of apoptosis in the treatment groups relative to the control group. The apoptosis ratios were significantly greater in SK-BR-3 cells (**a**, *P < 0.05) and the BT-474 cells (**b**, **P < 0.01), but not the JIMT-1 cells (**c**)

In order to assess the anti-proliferation effect of M802 to HER2-positive cancer cells in vitro, we performed proliferation assay using breast cancer cell lines SK-BR-3, BT-474 and JIMT-1. Previous studies suggested that Herceptin plays a role in suppressing the growth of HER2-overexpressing breast cancer cells [36]. Our results indicated that M802 also showed obvious dose-dependent effect on growth inhibition of SK-BR-3 cells (Fig. 4a, a) and BT-474 cells (Fig. 4a, b), but the effect was weaker than Herceptin. After day-4, the growth inhibition of M802 gradually became apparent. As expected, both M802 and Herceptin had no effect on the growth inhibition of HER2-resistant JIMT-1 cells (Fig. 4a, c). These results suggest that M802 retained part of Herceptin’s function, although it only had half of its structure.

The induction of apoptosis was evaluated using Annexin V/PI double labeling assay by flow cytometry (Additional file 1: Figure S2), and then determined the fold-change of apoptosis in the treatment groups to control group (Fig. 4b). M802 markedly increased the apoptotic ratio of SK-BR-3 and BT-474 cells compared with the control group, especially at high concentration (Fig. 4b, a and b). Although the apoptotic effect of Herceptin was stronger than that of M802, these two antibodies showed no statistically significant differences in the induction of apoptosis in JIMT-1 cells (Fig. 4b, c).

### M802 regulated PI3K/AKT and MAPK signal pathways

Since M802 had a role in inhibiting proliferation and promoting apoptosis in Herceptin-sensitive (HER2-positive) tumor cells, we examined the effect of M802 and Herceptin on changes in the levels of proteins in the classical pathways downstream of HER2 that regulate cell cycle progression and/or cell death after M802 and Herceptin administration.

**Fig. 5:**
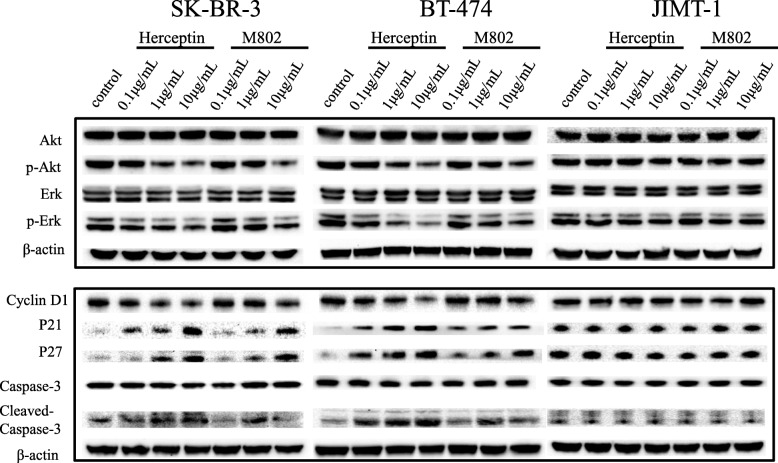
Effects of M802 on cancer-related signaling pathways. Cells were treated with M802 or Herceptin, and then harvested and subjected to western blotting for detection of Akt, p-Akt, Erk, and p-Erk after 24 h, and detection of cyclin D1, P21, P27, caspase-3, and cleaved-caspase-3 after 48 h. β-actin was used as the internal reference, and 3 independent replicates were performed

Analysis of SK-BR-3 and BT-474 cells indicated that Herceptin and M802 had significant dose-dependent effects on inhibition of the phosphorylation of Erk and Akt (but not total Erk or total Akt expression), down-regulation of cyclin D1, and up-regulation of p27 and p21 (Fig. 5). M802 and Herceptin also increased the level of the pro-apoptotic protein cleaved-caspase-3. Consistent with our previous results (Fig. 4), M802 and Herceptin treatment had no effect on the levels of p-Erk and p-Akt in JIMT-1 cells. These results thus indicate that the M802-mediated regulation of the PI3K/AKT and MAPK pathways are responsible for its effects on induction of apoptosis and cell cycle arrest.

### M802 activated T cells and induced the secretion of cytokines in vitro

**Fig. 6:**
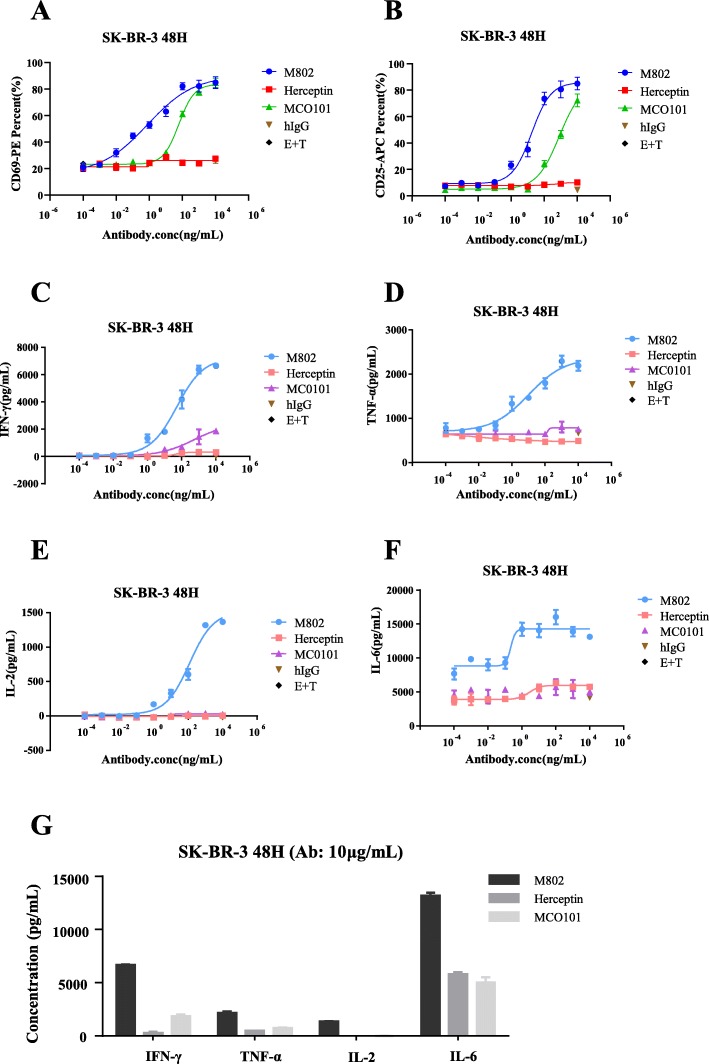
M802 activated T cells and induced the secretion of cytokines in vitro. (**a** and **b**) SK-BR-3 cells were incubated with PBMCs at an E:T ratio of 5:1 and with different concentrations of M802 or Herceptin or MCO101 for 48 h. The PBMCs were collected and stained with anti-CD3-FITC and anti-CD69-PE or anti-CD25-APC antibodies. The activation was determined by flow cytometry. The cell free supernatant was used to detect the cytokine release including IFN-γ (**c**), TNF-α (**d**), IL-2 (**e**) and IL-6 (**f**) by ELISA. (**g**) Secretion of cytokines by PBMCs induced by 10 μg/mL M802 and control antibodies. M802 significantly promoted the secretion of cytokines, especially IL-6 and IFN-γ

In addition to studying the mechanism of action of M802 at the anti-HER2 moiety, we also evaluated how the anti-CD3 moiety functions. The levels of CD25 and CD69, signs of T-cell activation, significantly increased in the killing system containing M802, target cells and effector cells (Fig. 6a-b). MCO101 also induced potent activation of T cells at high concentrations, possibly because of its anti-CD3 moiety and its intact Fc moiety. The cytokines released by effector cells in cell-free supernatants were measured by ELISA. And the results revealed that M802 induced robust secretion of IFN-γ (Fig. 6c), TNF-α (Fig. 6d), IL-2 (Fig. 6e), and IL-6 (Fig. 6f). We compared the secretion levels of individual cytokines when treated with 10 μg/mL of M802. The concentration of IL-6 was the highest, close to 15,000 pg/mL (Fig. 6g).

### Growth inhibition of tumors by M802 in vivo and pharmacokinetics study

**Fig. 7:**
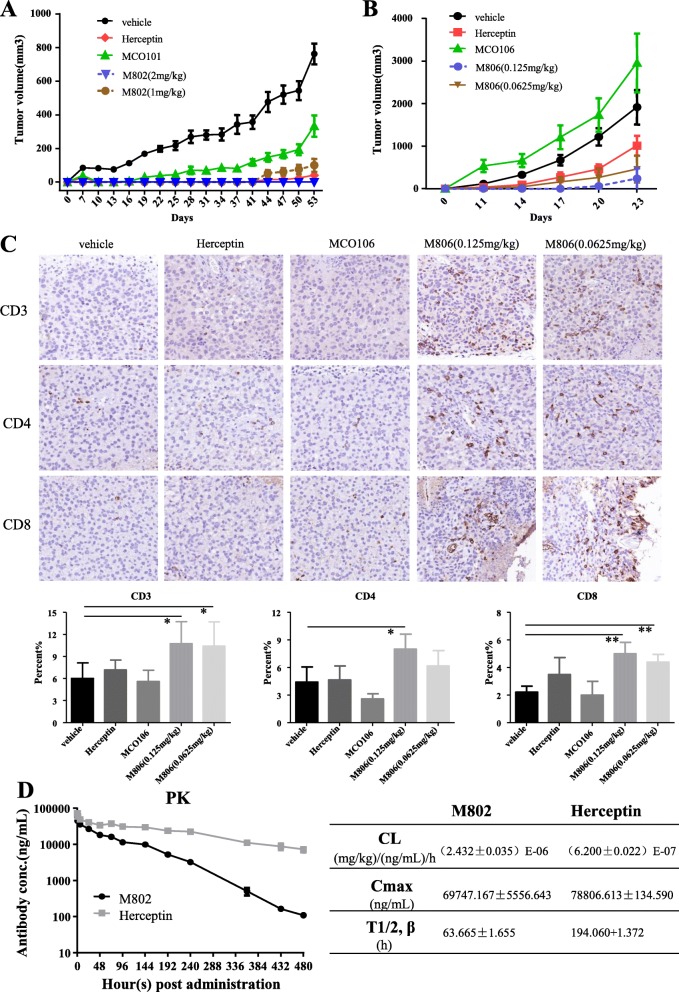
Effects of M802 and M806 on tumor growth in vivo. (**a**) Prophylactic treatment of NCI-N87 tumors in a subcutaneous xenograft model in NOD/SCID mice reconstituted with human CIKs. (**b**) Prophylactic treatment of B16-HER2 tumors in a syngeneic model in C57BL/6 mice which were subcutaneously inoculated with 3.0 × 10^6^ B16-HER2 cells. Data indicate mean tumor volumes ± SD. (**c**) Isolated tumor tissues were stained with anti-mouse CD3 antibody or anti-mouse CD4 antibody or anti-mouse CD8 antibody and analyzed by IHC. We calculated the proportion of CD3-, CD4-, and CD8-positive cells in the total cells and selected a representative image (20×) from each group. (**d**) PK profile of M802. A single dose (8 mg/kg) of Herceptin (*n* = 6) or M802 (n = 6) was intravenously injected into BALB/C mice. Serum samples were collected at different timepoints and detected by ELISA. Values indicate means ± SD. *P < 0.05, **P < 0.01

The in vivo antitumor efficacy of M802 was evaluated in an adoptive transfer xenograft model. The gastric cancer cell line NCI-N87 was used to establish xenograft tumor models in immunodeficient NOD/SCID mice. Human CIK cells were mixed with tumor cells at a ratio of 1:1 before inoculation into mice. In this study, compared with the control groups, both 2 mg/kg and 1 mg/kg M802 treatment groups showed significant inhibitory effects on tumor growth. Especially, 2 mg/kg M802 resulted in complete inhibition of tumor growth during the entire observation period of 53 days. In the group of 1 mg/kg M802, small nodules were observed during the later period. The control antibody MCO101 only showed a partial inhibition of tumor growth at the dose of 4 mg/kg. The inhibitory effects of 4 mg/kg Herceptin was similar to that of 1 mg/kg M802 (Fig. 7a).

In order to study the immune mechanism of M802 in vivo, we established another mice model. B16-HER2 cells were inoculated subcutaneously into C57BL/6 mice. M806 and other control antibodies were administrated according to the experimental design. M806 targeting human HER2 and mouse CD3 was an alternative molecule for M802. Our examination of the characteristics of M806, including purity, concentration, affinity and cytotoxicity in vitro, indicated it was suitable for these experiments (Additional file 1: Figure S3). M806 showed superior efficacy in inhibiting tumor growth during the 23-day observation period (Fig. 7b). The IHC results showed that the numbers of CD3-, CD4-, and CD8-positive cells in the M806 groups was obviously higher than that in the other control groups (Fig. 7c). M806 significantly increased the number of Tumor Infiltrating Lymphocytes (TILs). Before and after administration of antibodies, we also collected the peripheral blood of mice to detect changes of lymphocytes including B cells, CD4^+^ T cells and CD8^+^ T cells. The results indicated that the number of T cells in the peripheral blood decreased after each administration, mainly caused by decreased CD4^+^ T cells (Additional file 1: Figure S4). This phenomenon only occurred in groups of M806 and MCO106, thus demonstrating that the anti-CD3 moiety interacts with T lymphocytes.

Our pharmacokinetics study indicated that M802 had a more rapid systemic clearance (Cl) than Herceptin and a shorter serum half-life than Herceptin (64 h vs. 194 h) (Fig. 7d). The smaller Mw is a reason for the shorter half-life of M802. Moreover, the anti-CD3 moiety of M802 is a single chain unit based on the scFv structure, which has weaker stability than the intact IgG antibody. Therefore, at later timepoints, the detected concentrations of M802 by the double-antigen sandwich ELISA may be lower than the actual serum concentrations.

## Discussion

BsAbs have numerous potential clinical applications, including neutralization of different pathogenic mediators, recruitment of immune cells to tumor cells, and regulation of cell signaling [37]. However, the development of a BsAb as a therapeutic requires a platform that produces an antibody that is stable and has favorable pharmacokinetics, and that can be scaled up for commercial manufacturing. This kind of platform has rarely obtained up to now [38–40].

In the present study, we generated a new format of an anti-HER2 × anti-CD3 BsAb, M802, using the technologies of KIHs and salt bridges to engineer the CH3 domains. M802 contains two units, a monovalent unit and a single chain unit. The monovalent unit contains a heavy chain and a light chain that bind to HER2, and are structurally identical to Herceptin. The single chain unit is a BiTE format of L2K mAb fused with a human Fc fragment, and binds to CD3 [10, 41]. M802 has excellent thermal stability (Additional file 1: Table S3). Presently, M802 has been produced through three-strep wised chromatography and the purity was more than 98% (Fig. 1c). The theoretical Mw was 127 KDa, but the measured Mw was about 130 KDa since it contained two N-linked glycans at two glycosylation sites.

Our study indicated that M802 had several unique properties. First, the T366 W/K392D/K409D and L368R/D399K/D407A mutant pairs at the CH3 domain interface efficiently promoted heterodimerization, so that the heterodimer form was the predominant product in the cell culture supernatant.

Second, the purification of M802 was no longer a barrier because the Mw and pI were different for heterodimers and homodimers. In addition, M802 showed excellent thermal stability (Additional file 1: Table S2) and long half-life (Fig. 7d).

Third, compared with parent monoclonal antibodies, single chain unit of M802 retained relatively weaker binding capacity to CD3, but the affinity of monovalent unit of M802 was closer to Herceptin (Additional file 1: Table S3). The reduced affinity of single chain unit to CD3 was an advantage of M802, since activation of T cells should not be too strong in order to reduce the potential side effects of “cytokine-release storm”. Even though the affinity of M802 to CD3 was reduced (Kd = 186.8 nM), M802 was still sufficiently cytotoxic against tumor cells. This is similar to the BiTE molecule of CD19/CD3 which had a Kd of only 260 nM to T cells, but exhibited strong therapeutic effects [42].

Fourth, cancer cell lines that express high levels of HER2 (SK-BR-3, BT-474, and NCI-N87 cells), and Herceptin-resistant cancer cell line (JIMT-1) were much more sensitive to M802 than Herceptin when using PBMCs as effector cells (Fig. 3b to f). It was reported that JIMT-1 cells were resistant to Herceptin as the result of the coverage of HER2 binding site by MUC4 [43], so JIMT-1 could be regarded as a cell line with low level of HER2. M802 also mediated effector cells to lyse MDA-MB-231 cells at high concentration because of the cross-reactivity between M802 and EGFR expressed on the cells. Our results suggested that M802 could be more efficient than Herceptin for clinical applications due to its ability to simultaneously bind HER2-positive tumor cells and T lymphocytes. In contrast, M802 did not bind to or lyse HER2-negative non-tumor cells (HEK-293; Fig. 3g).

Finally, considering the monovalent unit of M802 was from Herceptin, we tested whether its anti-HER2 moiety had tumor suppressing effects. The results showed that M802 inhibited the proliferation and promoted the apoptosis of tumor cells, although its effects were weaker than Herceptin. As predicted, M802 had no effect on Herceptin-resistant JIMT-1 cells (Fig. 4). In addition, M802 and Herceptin had similar effects on downstream tumorigenesis signaling pathway proteins (Fig. 5). Besides, M802 recruited T cells to target HER2-positive tumor cells and induced HER2-dependent T-cell activation (Fig. 6a) and cytokine release (Fig. 6b to e). Therefore, M802 not only retained the functions of Herceptin on tumorigenesis signal pathways, but also recruited and activated CD3-positive T cells to eliminate tumor cells.

In animal model, M802 exhibited superior efficacy on inhibition of human gastric cancer cells (NCI-N87) in NOD-SCID mice compared with control antibodies (Fig. 7a). Since we artificially mixed effector cells with tumor cells in this experiment, the ability of M802 to recruit effector cells from immune system to kill tumor cells remains to be studied. Therefore, we performed another in vivo study using alternative BsAb M806 targeting human HER2 and murine CD3 on immunocompetent C57BL/6 mice. The results showed that M806 (0.125 and 0.0625 mg/kg) significantly inhibited the growth of B16-HER2 tumors in vivo (Fig. 7b) and recruited T lymphocytes to tumor tissues (Fig. 7c).

## Conclusions

Our studies demonstrated that the BsAb M802, developed using KIHs, salt bridges, and an asymmetrical format, could be produced with high quality. At present, M802 has been approved for phase I clinical trial for patients with HER2-positive advanced breast cancer by National Medical Products Administration (NMPA) (2017 L04744). MSBODY antibodies are active in vivo and have pharmacokinetic properties that are comparable with those of regular human monoclonal antibodies. In addition, the BsAb platform is generally suitable for different targets, and therefore, a variety of MSBODY can be constructed. Thus, the MSBODY technology may be an effective way to produce immunotherapeutic BsAbs for the treatment of cancers or other human diseases.

## Additional file

Additional file 1: **Figure S1.** The expression level of HER2 on cell surface. **Figure S2.** The flow cytometry figures of apoptosis. **Figure S3.** The quality test results of M806. **Figure S4.** The changes of lymphocytes in peripheral blood. **Table S1.** Primers used for construction of the BsAb. **Table S2.** Thermal challenge assay of BsAb. **Table S3.** Affinity measurements of antibodies. (PDF 645 kb)

## Data Availability

All data generated or analyzed during this study are included in this article or in the supplementary files.

## Abbreviations

ADCC: Antibody-dependent cell-mediated cytotoxicity
BiTE: Bispecific T-cell engager
BSA: Bovine serum albumin
BsAb: Bispecific antibody
CCTCC: China Center for Type Culture Collection
cFAE: Controlled Fab-arm exchange
CIK: Cytokine-induced killer
CL: Systemic clearance
Cmax: Maximum serum concentration
DART: Dual-affinity re-targeting
DSMZ: Deutsche Sammlung von Mikroorganismen und Zellkulturen
FDA: Food and Drug Administration
HER2: Human epidermal growth factor receptor 2
HPLC-SEC: high-performance liquid chromatography size-exclusion chromatography
NMPA: National Medical Products Administration
PBMCs: Peripheral blood mononuclear cells
PK: Pharmacokinetic
PVDF: Polyvinylidene fluoride
RP-HPLC: Reverse-phase high performance liquid chromatography
SDS-PAGE: Sodium dodecyl sulfate-polyacrylamide gel electrophoresis
SEED: Strand exchange engineered domain
SPR: Surface plasmon resonance
TILs: Tumor infiltrating lymphocytes
