# Supplementary material for: A novel asymmetrical anti-HER2/CD3 bispecific antibody exhibits potent cytotoxicity for HER2-positive tumor cells
Source: J Exp Clin Cancer Res. 2019 Aug 14;38:355. doi: 10.1186/s13046-019-1354-1 (PMC6694677; doi:10.1186/s13046-019-1354-1)
Supplement: Supplementary file 1 — Figure S1. The expression level of HER2 on cell surface. Figure S2. The flow cytometry figures of apoptosis. Figure S3. The quality test results of M806. Figure S4. The changes of lymphocytes in peripheral blood. Table S1. Primers used for construction of the BsAb. Table S2. Thermal challenge assay of BsAb. Table S3. Affinity measurements of antibodies. (PDF 645 kb) [file 13046_2019_1354_MOESM1_ESM.pdf]

Supplementary Figure S1. The expression level of HER2

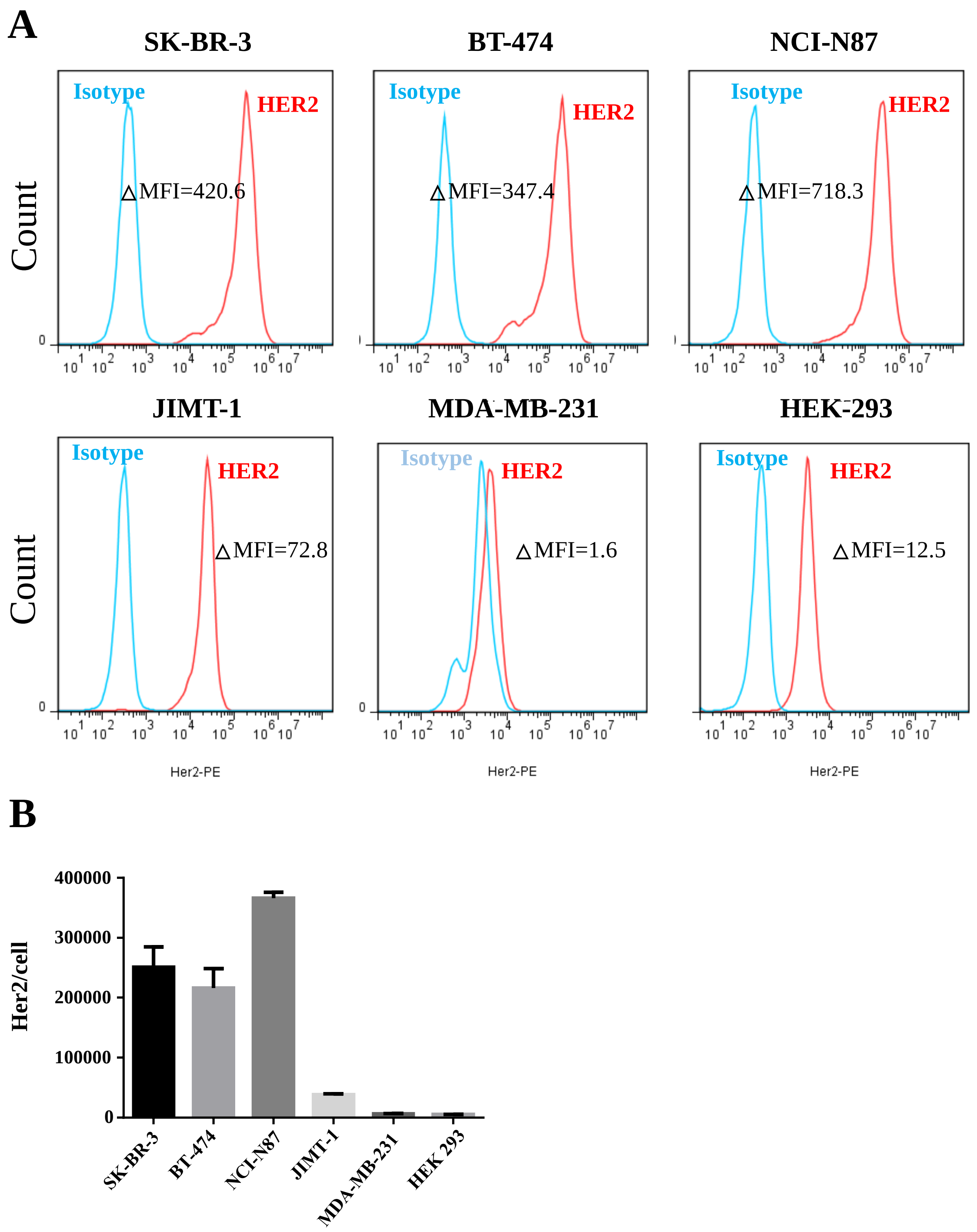

Supplementary Figure S1. The expression level of HER2 on cell surface.
We used PE-labeled anti-human HER2 fluorescent antibody and QuantibriteTM Beads PE Fluorescence Quantitation Kit (BD) detected the expression level of HER2 on different cells surface according to the manufacture's protocol. (A) Mean fluorescence intensity (MFI) of HER2 and isotype on different cells were calculated by FlowJo software (Version 7.6). (B) Number of HER2 molecules on per cell surface. ΔMFI=MFI (HER2)/MFI (isotype).

Supplementary Figure S2. The flow cytometry figures of apoptosis

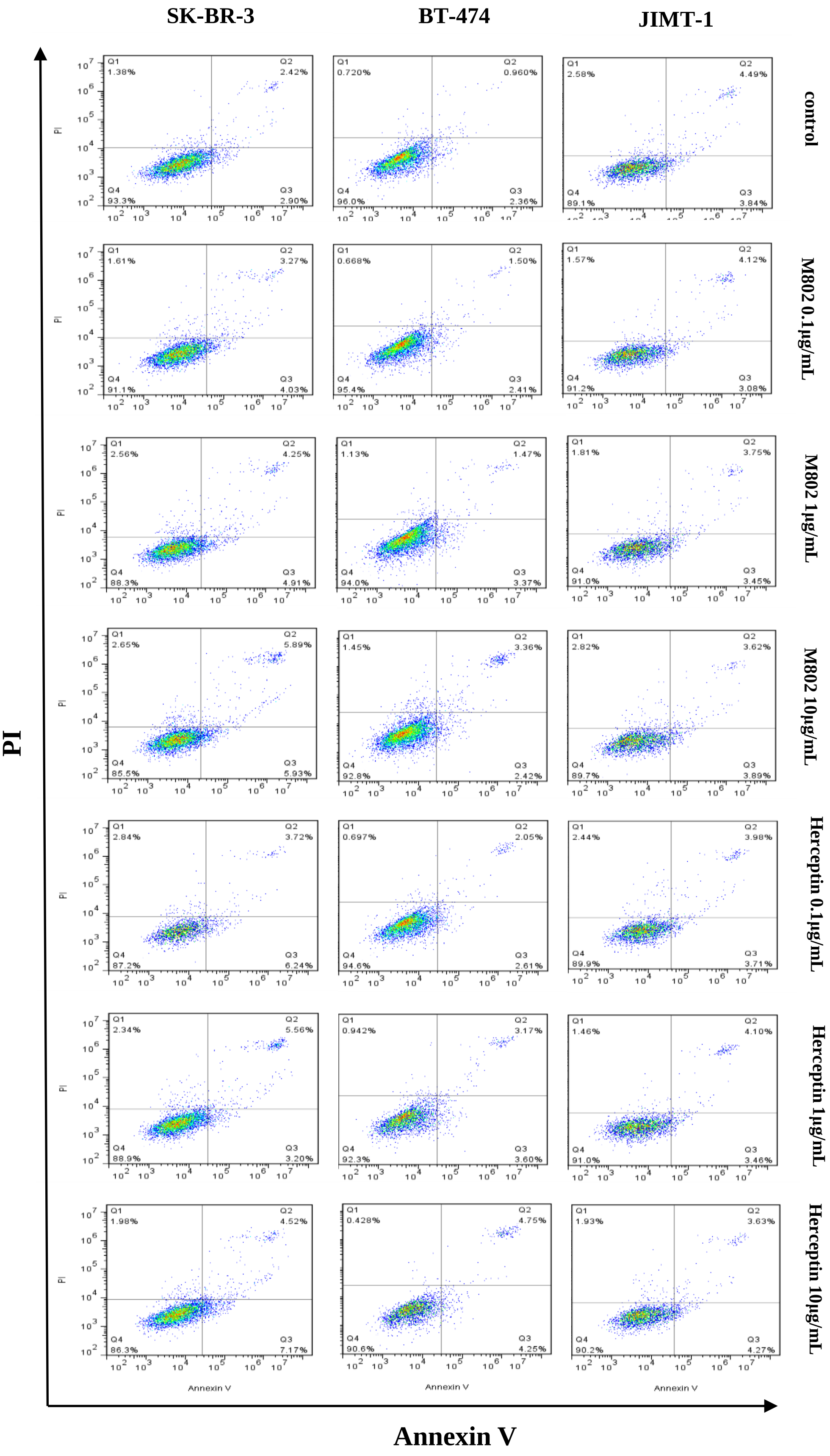

Supplementary Figure S2. The flow cytometry figures of apoptosis.
SK-BR-3 cells, BT-474 cells and JIMT-1 cells were treated with M802 (0.1, 1, 10μg/mL) and Herceptin (0.1, 1, 10μg/mL) for 48 hours, respectively. Collected cells were stained with Annexin V/PI and detected by flow cytometry.

Supplementary Figure S3. The quality test results of M806

A

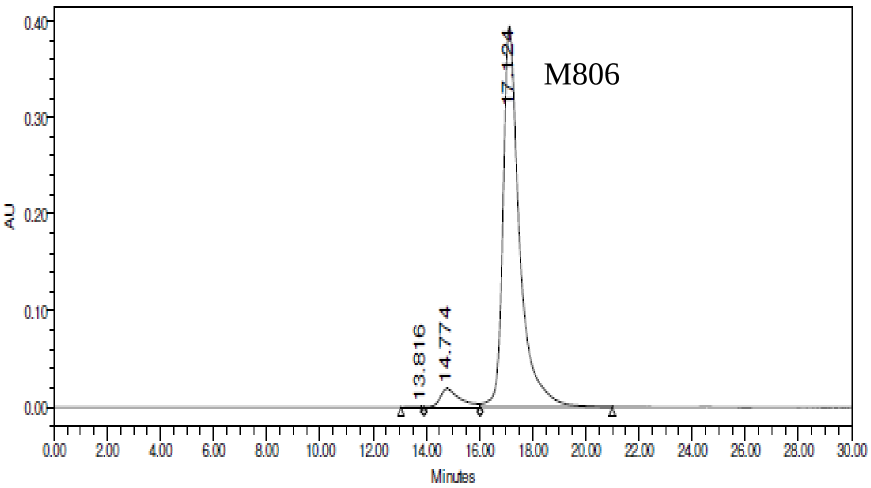

B

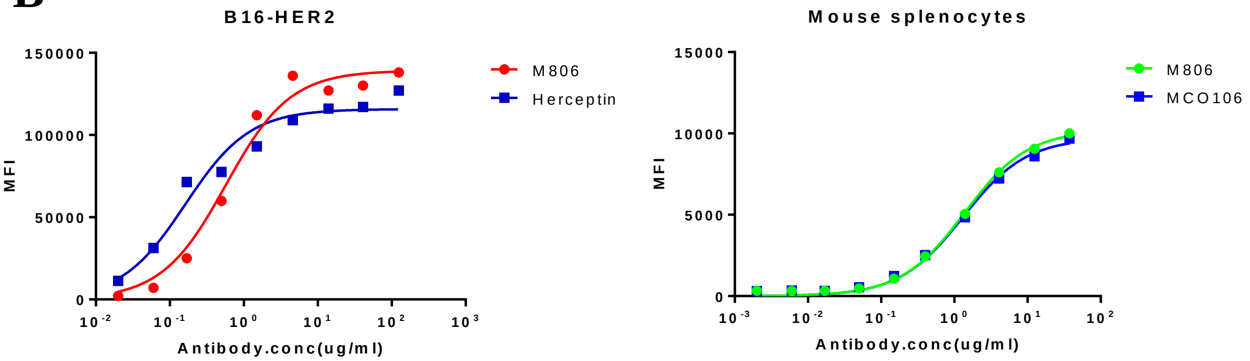

	M806	Herceptin
Kd	0.5719	0.1622

	M806	MCO106
Kd	1.372	1.311

C

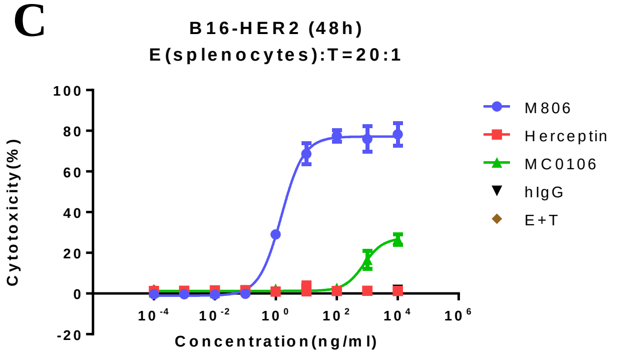

	M806	Herceptin	MC0106
Top	77.16		27.27
LogEC50	0.1843		2.890
HillSlope	1.190		1.384
EC50	1.529		776.6

Supplementary Figure S2. The quality test results of M806.

(A) The purity of M806 was 94.51%. (B) The cell binding activity of M806 on anti-HER2 end and anti-CD3 end. (C) M806 efficiently induced effector cells (mouse splenocytes) to kill target cells (B16-HER2 cells) in vitro.

Supplementary Figure S4. The changes of lymphocytes *in vivo*

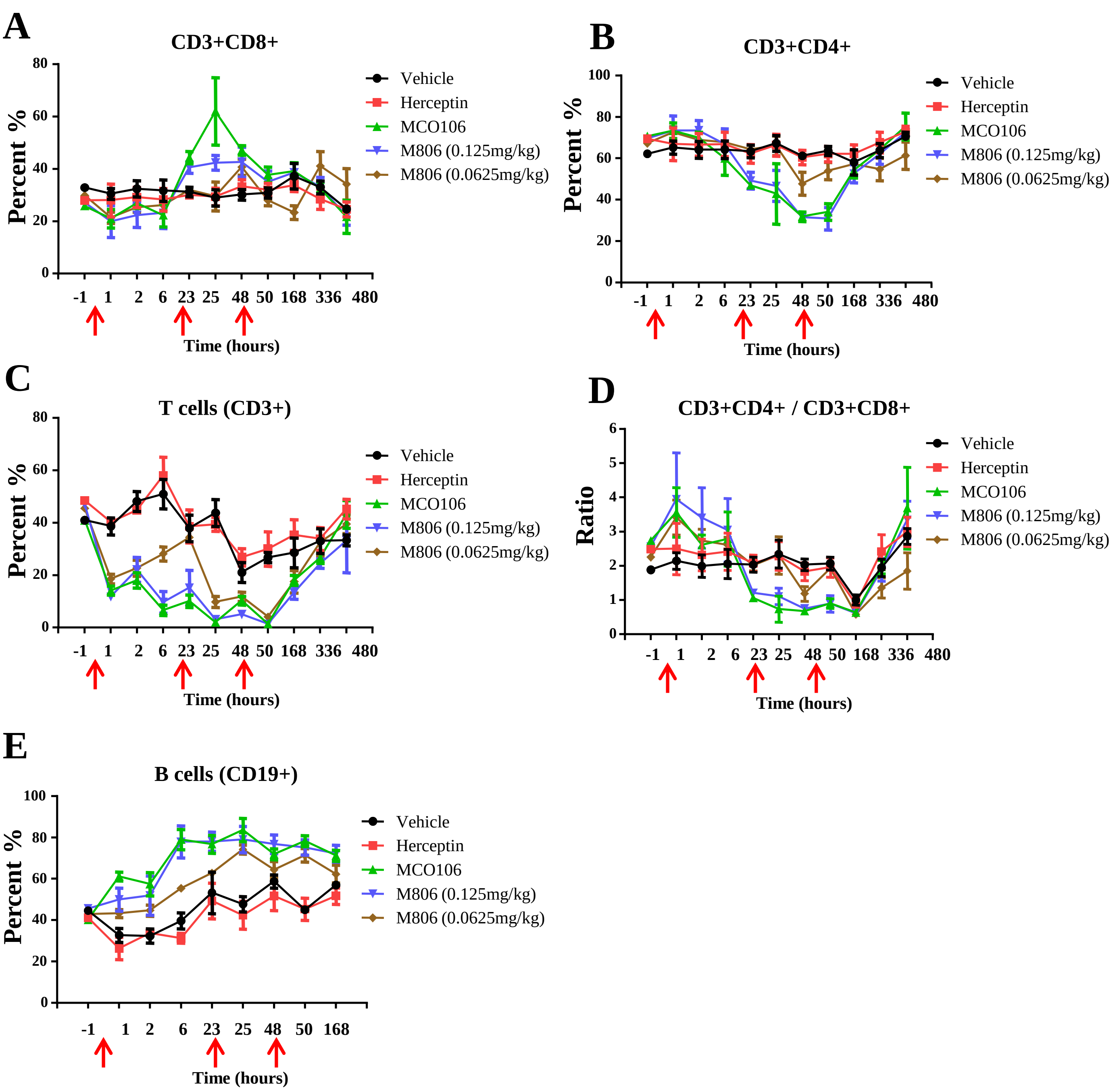

Supplementary Figure S3. The changes of lymphocytes in peripheral blood.
Before and after administration of antibodies, we extracted the peripheral blood of mice to detect changes of lymphocytes. (A) Percentage of CD3+CD8+ cells/lymphocytes (B) Percentage of CD3+CD4+ cells/lymphocytes (C) Percentage of T cells (CD3+)/lymphocytes (D) The ratio of CD3+CD4+ cells/ CD3+CD8+ cells (E) Percentage of B cells (CD19+)/lymphocytes. Arrow: time points of administration.

Supplementary Table S1. Primers used for construction of the BsAb

**Primer**	**Sequence**	**Note**
Leader1-F	CGCGAATTCGATATCGCCACCATGGAGACAGACACACTCCTGCTA	Leader peptide-1 5’
Leader1-R	CTGTGGAACCTGGAACCCAGAGCAGCAGCACCCATAGCAGGAGTGTGTCT	Leader peptide-1 3’
Leader2-F	CGCGAATTCGATATCGCCACCATGGAAACGGATACGCTACTCCTC	Leader peptide-2 5’
Leader2-R	CCGTCGACCCGGGCACCCATAGCAGCAGCACCCAGAGGAGTAGCGTATCC	Leader peptide-2 3’
Fc-F	GGTGCGGCCGCAGAGCCCAAATCTTGTGACAAAAC	Human IgG1 Fc 5’
Fc-R	GCGTCTAGACTCGAGTCATTTACCCGGAGACAGGGAGAGGC	Human IgG1 Fc 3’
HerVH-F	ATGGGTGCCCGGGTCGACGGGGGAAGTGCAGCTGGTGGAAAGCG	Herceptin heavy chain VH 5’
HerVL-F	CTGGGTTCCAGGTTCCACAGGTGATATTCAGATGACCCAGAGCC	Herceptin light chain VL 5’
CK-F	TAAACGCACCGTGGCGGCGCCGAGC	Human Ig kappa chain CL 5’
CK-R	CGAGCTCGGATCCTTAGCATTCGCCGCGGTT	Human Ig kappa chain CL 3’
L2KVH-F	ATGGGTGCCCGGGTCGACGGGGGATATCAAACTGCAGCAGTCAG	L2K VH 5’
L2KVH-R	GCCTGAACCGCCGCCTCCTGAGGAGACTGTGAGAGTG	L2K VH 3’
L2KVL-F	AGTGGTGGAGGAGGTTCTGACATTCAGCTGACCCAGTCTC	L2K VL 5’
L2KVL-R	GGGCTCTGCGGCCGCACCTTTCAGCTCCAGCTTGGTC	L2K VL 3’
Linker-F	GGAGGCGGCGGTTCAGGCGGAGGTGGAAGTGGTGGAGGAGGTTCT	Soft linker 5’
Linker-R	AGAACCTCCTCCACCACTTCCACCTCCGCCTGAACCGCCGCCTCC	Soft linker 3’
T366W-F	ACCAGGTCAGCCTGTGGTGCCTGGTCAAA	Fc/T366W 5’
T366W-R	TTTGACCAGGCACCACAGGCTGACCTGGT	Fc/T366W 3’
L368R-F	GTCAGCCTGACCTGCCGGGTCAAAGGCTTCTAT	Fc/L368R 5’
L368R-R	ATAGAAGCCTTTGACCCGGCAGGTCAGGCTGAC	Fc/L368R 3’
K392D-F	GGAGAACAACTACGATACCACGCCTCCCGT	Fc/K392D 5’
K392D-R	ACGGGAGGCGTGGTATCGTAGTTGTTCTCC	Fc/K392D 3’
D399K-F	CGCCTCCCGTGCTGAAGTCCGACGGCTCCTTC	Fc/D399K 5’
D399K-R	GAAGGAGCCGTCGGACTTCAGCACGGGAGGCG	Fc/D399K 3’
Y407A-F	TCCTTCTTCCTCGCCAGCAAGCTCACCGT	Fc/Y407A 5’
Y407A-R	ACGGTGAGCTTGCTGGCGAGGAAGAAGGA	Fc/Y407A 3’
K409D-F	CTTCCTCTACAGCGATCTCACCGTGGACA	Fc/K409D 5’
K409D-R	TGTCCACGGTGAGATCGCTGTAGAGGAAG	Fc/K409D 3’

The restriction endonuclease sites are underlined.

Supplementary Table S2. Thermal challenge assay of BsAb

	$T_{50\text{-HER2}}$(°C)	$T_{50\text{-CD3}}$(°C)
Herceptin	61.26	N/A
M802	60.71	57.21
L2K	N/A	59.95

Data was analyzed with Graphpad prism 6 to calculate the temperature (T_{50}) at which 50% of antibodies retained binding to cells following thermal challenge. The $T_{50\text{-HER2}}$ referred to antibodies binding to SK-BR-3 cells, and the $T_{50\text{-CD3}}$ referred to antibodies binding to Jurkat cells.

Supplementary Table S3. Affinity measurements of antibodies

	k_a, **1/Ms**	k_d, **1/s**	KD, **M**
M802 and HER2 interaction	(4.29±0.17) E+05	(2.48±0.15)E-04	(5.78±0.12)E-10
Herceptin and HER2 interaction	(1.12±0.035) E+06	(1.27±0.23)E-04	(1.14±0.23)E-10
M802 and CD3 interaction	(3.45±0.191) E+05	(2.45±0.18)E-02	(7.12±0.91)E-08
L2K and CD3 interaction	(1.07±0.072) E+07	(1.31±0.19)E-02	(1.23±0.18)E-09

The association rate constant (ka) and the dissociation rate constant (kd) were measured with PriteOn. The equilibrium dissociation constant KD was calculated as KD = Kd/Ka.
